# Data With Dialogue: Empowering Nursing Home Teams Through Small Data Reports

**DOI:** 10.1093/geroni/igaf122.2064

**Published:** 2025-12-31

**Authors:** Vanessa Aguilar, Brian Cox, Hope Carwile, David Farrell, Jennie Keleher, Catherine McGuire, Angela Westergard, A Lynn Snow

**Affiliations:** The University of Alabama, Tuscaloosa, Alabama, United States; Alabama Research Institute on Aging, Tuscaloosa, Alabama, United States; Vivage, Lakewood, Colorado, United States; The University of Alabama, Tuscaloosa, Alabama, United States; The University of Alabama, Tuscaloosa, Alabama, United States; Caraday Health, San Marcos, Texas, United States; National HealthCare Corporation, Murfreesboro, Tennessee, United States; The University of Alabama, Tuscaloosa, Alabama, United States

## Abstract

40Winks, a pragmatic trial aimed at improving nighttime sleep quality and daytime activity among nursing home residents, leverages wearable technology to translate small data into actionable insights. Individual resident data from wrist actigraphy devices is compiled into a care team report carefully designed for easy interpretation by non-researchers. The report visually represents residents’ 24-hour sleep, activity, and light exposure across seven days. This helps nursing home staff identify both trouble areas for intervention and bright spots where care is succeeding. The report is specifically designed to be used in whole team discussions that include front-line staff, fostering dialogue and collaboration across role and department silos. The reports aid the whole team in intervention planning to minimize nighttime sleep disruptions (e.g., light or noise) and increase daytime light exposure and activity. By providing an accessible visual and easily understandable and digestible amounts and types of data, the whole team including front-line staff can pinpoint areas needing attention while also recognizing effective care practices. For residents undergoing multiple assessments, these reports provide a concrete way for teams to track progress, test and refine interventions, and sustain successful strategies over time. Training nursing home teams on small data and how to use it for powerful team dialogue enhances the quality of resident care, provides concrete evidence for the effectiveness of trialed interventions, and supports implementation of resident-centered care improvements over time. By integrating small data into daily practice, nursing home teams can drive continuous improvement, leading to sustained enhancements in resident-centered care.

